# Examining patterns of assistive device distribution under the Indian ADIP scheme from 2020 to 2025

**DOI:** 10.3389/fresc.2026.1848396

**Published:** 2026-08-19

**Authors:** S. Nisha, J. Balamurugan

**Affiliations:** Department of Social Sciences, School of Social Sciences and Languages, Vellore Institute of Technology, Vellore, India

**Keywords:** ADIP scheme, assistive devices, beneficiary coverage, interstate disparities, persons with disabilities, program implementation patterns

## Abstract

Access to assistive devices is essential for the rehabilitation and social participation of persons with disabilities and constitutes one of the major development strategies for persons with disabilities in India. The Assistance to Disabled Persons for Purchase/Fitting of Aids and Appliances (ADIP) scheme was introduced by the Ministry of Social Justice and Empowerment, Government of India as an initiative to support economically disadvantaged people with disabilities in procuring such devices. Despite its national mandate, there is limited literature on how the scheme is implemented across Indian states. This study examines interstate variation in the implementation of the ADIP scheme in India over 5 years, from 2020 to 2025. Using secondary data from publicly available sources, three indicators were constructed: beneficiary coverage relative to the disabled population, fund utilization per beneficiary, and beneficiaries served per distribution camp. To assess the degree of interstate inequality, descriptive statistical methods, including coefficient of variation and range ratio, were applied. The findings reveal differences in fund utilization and camp outreach, highlighting uneven implementation of the scheme. The results indicate that access to assistive devices under the ADIP scheme is geographically spread unequally across Indian states and Union Territories (UTs). Therefore, this study advocates for more balanced, region-specific implementation strategies to ensure equity in beneficiary coverage.

## 1 Introduction

According to the 2011 Census of India, there are 26.8 million persons with disabilities in the country, representing 2.21% of the total population (1). Access to assistive devices is essential for the rehabilitation and social participation of persons with disabilities, providing a means to access educational, vocational, social, and recreational opportunities (2). The use of assistive devices promotes functional abilities and improves community participation. Active community participation contributes to personal wellbeing and the development of an inclusive society (3). Assistive devices such as mobility aids, hearing devices, and visual aids help individuals improve independent functioning, help overcome functional limitations, and mitigate the extent of disability. The World Health Organization and UNICEF recognize access to assistive technology as a crucial component of inclusive health systems for persons with disabilities (4). Research further emphasizes that assistive technology contributes to enhanced independence, improved quality of life, and greater social inclusion (5). Improving access to assistive technology can contribute to achieving the Sustainable Development Goals and ensures that no one is left behind (6).

The Global Report on Assistive Technology estimates that more than 2.5 billion people worldwide need assistive products, yet a large proportion of the needy lack access to appropriate devices (4). This gap is particularly pronounced in developing and underdeveloped countries, where service delivery systems are often weak. Khasnabisa et al. (5) and MacLachlan et al. (7) identified that challenges such as unaffordability, limited service infrastructure, and lack of trained personnel restricted access to assistive devices in certain regions. In addition, many people with disabilities and their families remain unaware of available products and services and their developmental dimensions (8). The usability and effectiveness of assistive technology is affected when the development of such technology does not always follow user needs and expectations (9). A study conducted across eight districts in India found that inability to afford assistive devices was reported as the most common barrier, affecting 36.9% of those with unmet needs (10). Furthermore, a global market report highlighted that market failures, including trade barriers and inefficient supply chains, caused price hikes. Hence, assistive products reach only 10% of those who need them, especially in low-income countries (11).

International mandates such as United Nations Convention on the Rights of Persons with Disabilities (UNCRPD) and the Incheon Strategy, along with national legislation like Rights of Persons with Disabilities Act, 2016, emphasize the role of assistive devices in providing accessibility and fostering independence for people with disabilities. The Government of India has undertaken several initiatives to implement these mandates. The National Institute for the Empowerment of Persons with Visual Disabilities (NIEPVD), under the Ministry of Social Justice and Empowerment, manufactures assistive devices for persons with visual impairment and distributes them at affordable cost (12). Similarly, the Artificial Limbs Manufacturing Corporation of India (ALIMCO) manufactures and supplies quality aids and appliances at subsidy rates to those in need (13, 14).

The Assistance to Disabled Persons for Purchase/Fitting of Aids and Appliances (ADIP) scheme is a major initiative launched by the Department of Empowerment of Persons with Disabilities, Government of India in 1981. The scheme aims to expand access to assistive devices (15) and help people with disabilities obtain durable, modern, and high-quality aids and appliances that support their physical, social, and emotional recovery by minimizing the impact of disabilities and improving their economic potential. Grants are given to various agencies—including the ALIMCO, national institutes, composite regional centers, district disability rehabilitation centers, state corporations focused on disability, and NGOs—for the purchase and distribution of aids and assistive devices. This scheme also creates awareness about assistive devices among people with disabilities and their organizations. The Government of India organizes ADIP camps and distributes assistive devices to people with diverse disabilities across the country (15).

The implementation of the RPwD Act, 2016 in India has been hampered by bureaucratic inefficiencies, limited awareness, insufficient funding, and accessibility gaps, particularly in assistive technology provisioning schemes such as ADIP (16–18). More than half of persons with severe disabilities in India have unmet assistive technology needs, and among those who do obtain devices, nearly two-thirds purchase them out-of-pocket from private sources rather than through government schemes such as ADIP (10, 19, 20). Data from the National Sample Survey (NSS) and the Longitudinal Ageing Study in India (LASI) reveal sharp socioeconomic and regional disparities in assistive device use among persons with disabilities in India, reflecting underlying differences in institutional capacity, service infrastructure, and affordability across regions (21–23). A comparative analysis across South Asia reveals systemic weaknesses in AT service delivery, with poorly developed provisions across Nepal, India, and Bangladesh. These are marked by limited user awareness, restricted service availability, affordability barriers, and inadequate government funding (24–26).

Against this backdrop, the present study seeks to investigate how the ADIP scheme has been implemented across different states in India. The objective is to identify disparities that emerge at the regional level by focusing on three dimensions: beneficiary coverage, financial utilization, and outreach of distribution camps in each state and UT. Together, these dimensions offer a comprehensive descriptive picture of ADIP’s assistive device distribution across the country. Ultimately, a clearer understanding of where and why implementation varies can inform ongoing efforts to strengthen the reach, equity, and responsiveness of assistive device programs for persons with disabilities across India.

## 2 Materials and methods

This study uses secondary data from publicly available sources. Data on beneficiaries, funds utilized, and distribution camps under the ADIP scheme were obtained from the Indiastat database (Lok Sabha Unstarred Question no. 2613, dated 16 December 2025) and the Annual Report 2023–2024 of the Department of Empowerment of Persons with Disabilities (DEPwD). State-wise data on persons with disability were drawn from the 2011 Census of India. Beneficiary data are available for the Period 2020–2025, while data on fund utilization and camps are available from 2020 to 2024. Ladakh was excluded because of data unavailability across the study period, and Dadra and Nagar Haveli and Daman and Diu were treated as a single unit following their administrative merger.

Three indicators were constructed to examine program implementation, each derived using a simple ratio-based method appropriate for descriptive state-wise comparison. First, beneficiary coverage measures the outreach of the scheme relative to the disabled population and is calculated as follows: Beneficiary Coverage (%) = (Total ADIP beneficiaries, 2020–2025/Disabled population, Census 2011) × 100, computed separately for each state/UT. Second, fund utilization per beneficiary captures the average expenditure per person and is calculated as follows: Fund Utilized per Beneficiary = Total funds utilized/Total number of beneficiaries. Third, camp outreach captures the average number of persons served per distribution camp and is calculated as follows: Beneficiaries per Camp = Total beneficiaries/Total number of distribution camps organized. All three indicators were computed independently for each state and union territory using the secondary data described previously, allowing direct cross-state comparison without requiring additional modeling assumptions.

This study employs descriptive statistical methods to analyze interstate disparity. State-wise data are presented in tables and figures to enable comparison across indicators. To assess the extent of inequality across the three dimensions, two statistical measures are calculated for each indicator: coefficient of variation (CV), which indicates the degree of dispersion, and range ratio, which captures the gap between the highest and lowest values. The analysis is descriptive in nature, and findings are interpreted as patterns of variation between regions across the study period. Tables are manually compiled from the aforementioned sources, and graphs are generated with the assistance of Claude (Claude Sonnet 4.6, Anthropic).

## 3 Results

The data analysis is based on state-wise distribution of assistive devices under the ADIP scheme, implemented by the Department of Empowerment of Persons with Disabilities under the Ministry of Social Justice and Empowerment in India. The dataset compiles information on beneficiaries who received assistive devices across 36 states and union territories from 2020–2021 to 2024–2025.

### 3.1 Descriptive overview of ADIP beneficiaries

This descriptive overview provides a basis for examining patterns of program coverage and identifying disparities in access to assistive device across states and union territories. Table 1 presents an overview of the reach of the ADIP scheme during the period from 2020–2021 to 2024–2025.

**Table 1:** Overview of the assistance to disabled persons for purchase/fitting of aids and appliances (ADIP) scheme (2020–2021 to 2024–2025).

Indicator	Value
Total beneficiaries in India	1,235,669
Number of states and UTs covered	36
Average beneficiaries per state	34,324.13
State with the highest number of beneficiaries	Uttar Pradesh
State with the lowest number of beneficiaries	Lakshadweep
State with the highest beneficiary coverage relative to the disabled population	Dadra and Nagar Haveli and Daman and Diu
State with the lowest beneficiary coverage relative to the disabled population	Chhattisgarh
State with the highest funds utilized per beneficiary	Andhra Pradesh
State with the lowest funds utilized per beneficiary	Lakshadweep
State with the highest average number of beneficiaries per camp	Goa
State with the lowest average number of beneficiaries per camp	Sikkim

Lok Sabha unstarred question no. 2613, dated 16 December 2025) (31).

### 3.2 Interstate distribution analysis

To assess disparities in program outreach according to need, beneficiary coverage is calculated as the ratio of total ADIP beneficiaries to the total disabled population in each state.

**Figure 1:**
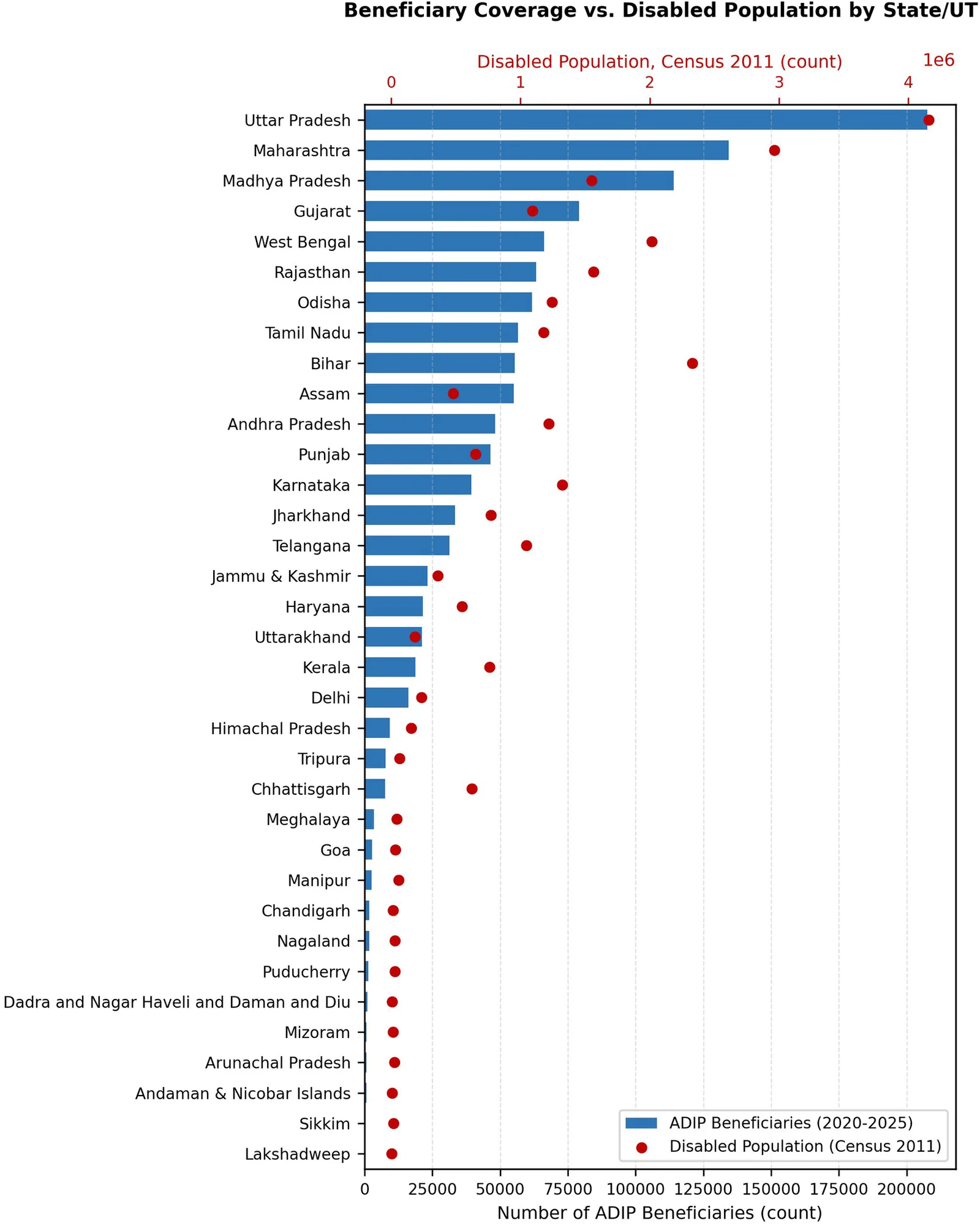
State-wise beneficiary coverage versus disabled population by state and UT. Source: Indiastat database (Lok Sabha unstarred question no. 2613, dated 16 December 2025) and Population Census 2011, Office of the Registrar General & Census Commissioner, India (32).

Table 2 presents the coverage of beneficiaries under the ADIP scheme relative to the population of persons with disabilities across states and union territories in India for the period 2020–2025. The data indicate significant variations in coverage levels across regions. Smaller states and union territories—including Dadra and Nagar Haveli and Daman and Diu, Lakshadweep, Chandigarh, Tripura, Assam, Uttarakhand, Andaman and Nicobar Islands, Goa, Meghalaya, Madhya Pradesh, Gujarat, and Punjab—demonstrate relatively high coverage compared with their disabled population. In contrast, large states such as Uttar Pradesh, Maharashtra, Rajasthan, and Tamil Nadu show moderate coverage levels. Lower levels of coverage are observed in states such as Chhattisgarh, Sikkim, Bihar Kerala, and Arunachal Pradesh, suggesting a very limited reach of beneficiaries relative to their population of disabled people. Figure 1 compares the number of ADIP scheme beneficiaries against the total disabled population according to the 2011 Census across all states and union territories in India.

**Table 2:** State-wise coverage of ADIP beneficiaries relative to the disabled population in India (2020–2025).

State/union territory	Total disability population (census 2011)	ADIP beneficiaries (2020–2025)	Beneficiaries’ coverage (%)
Andaman and Nicobar Islands	6,660	680	102.1
Andhra Pradesh	1,219,785	48,216	39.5
Arunachal Pradesh	26,734	715	26.7
Assam	480,065	55,036	114.6
Bihar	2,331,009	55,391	23.8
Chandigarh	14,796	1,795	121.3
Chhattisgarh	624,937	7,660	12.3
Dadra and Nagar Haveli and Daman and Diu	5,490	1,108	201.8
Delhi	234,882	16,140	68.7
Goa	33,012	2,827	85.6
Gujarat	1,092,302	79,126	72.4
Haryana	546,374	21,483	39.3
Himachal Pradesh	155,316	9,315	60
Jammu and Kashmir	361,153	23,181	64.2
Jharkhand	769,980	33,385	43.4
Karnataka	1,324,205	39,373	29.7
Kerala	761,843	18,726	24.6
Lakshadweep	1,615	272	168.4
Madhya Pradesh	1,551,931	113,964	73.4
Maharashtra	2,963,392	134,261	45.3
Manipur	58,547	2,534	43.3
Meghalaya	44,317	3,477	78.5
Mizoram	15,160	738	48.7
Nagaland	29,631	1,677	56.6
Odisha	1,244,402	61,777	49.6
Puducherry	30,189	1,385	45.9
Punjab	654,063	46,407	71
Rajasthan	1,563,694	63,393	40.5
Sikkim	18,187	323	17.8
Tamil Nadu	1,179,963	56,601	48
Telangana	1,046,822	31,311	29.9
Tripura	64,346	7,731	120.1
Uttar Pradesh	4,157,514	207,635	49.9
Uttarakhand	185,272	21,191	114.4
West Bengal	2,017,406	66,291	32.9

Indiastat database (Lok Sabha unstarred question no. 2613, dated 16 December 2025) and Population Census 2011, Office of the Registrar General & Census Commissioner, India (32).

The final dataset includes all states and union territories of India, with the exception of Ladakh, due to data unavailability. Dadra and Nagar Haveli and Daman and Diu are treated as a combined unit following their administrative merger. Beneficiary figures represent cumulative distributions during the study period (2020–2025). Coverage is calculated as the ratio of total ADIP beneficiaries to the total disabled population in each state based on the 2011 Census. Coverage values exceeding 100% may occur because beneficiary figures represent cumulative distributions during the study period, and some individuals may receive multiple assistive devices.

The coefficient of variation and range ratio are calculated to quantify the degree of variation in beneficiary coverage across states. The mean coverage is 64.7, with a standard deviation of 42.26, yielding a coefficient of variation of 0.65. The high variability indicates a wider spread in coverage levels, reflecting significant interstate inequality in access to assistive devices under the ADIP scheme. The range ratio of 16.4 further clarifies this disparity. The highest coverage is recorded in Dadra and Nagar Haveli and Daman and Diu (201.8%), which is 16 times greater than the lowest-performing state, Chhattisgarh (12.3%), highlighting the uneven geographic reach of the scheme.

### 3.3 State-wise fund utilization under the ADIP scheme

To examine the financial dimension of ADIP implementation, state-wise fund utilization is compared. Fund utilization per beneficiary is calculated as the average expenditure incurred per beneficiary.

Table 3 depicts the state-wise funds utilized under the ADIP scheme from 2020–2021 to 2023–2024. The results reveal variations in funds utilized per beneficiary across states and union territories in India. Andhra Pradesh records the highest fund utilization per beneficiary (₹12,900), followed by Telangana (₹12,600) and Haryana (₹12,100). Several smaller states such as Manipur and Sikkim also demonstrate a relatively high average utilization of funds. In contrast, comparatively low expenditure per beneficiary is observed in the states of Assam, Uttarakhand, and Himachal Pradesh and in the union territories of Dadra and Nagar Haveli and Daman and Diu and Lakshadweep. These results indicate that funds utilized for assistive device distribution differ widely across regions and are not aligned with beneficiary coverage.

**Table 3:** State-wise fund utilization per beneficiary under the ADIP scheme in India (2020–2024).

State/union territory	ADIP beneficiaries	Total fund utilized (INR million)	Fund utilized per beneficiary (INR)
Andaman and Nicobar Islands	680	2.61	3,800
Andhra Pradesh	48,216	621.65	12,900
Arunachal Pradesh	715	5.8	8,100
Assam	55,036	281.23	5,100
Bihar	55,391	519.29	9,400
Chandigarh	1,795	14.56	8,100
Chhattisgarh	7,660	62.32	8,100
Dadra and Nagar Haveli and Daman and Diu	1,108	2.97	2,700
Delhi	16,140	133.25	8,300
Goa	2,827	23.04	8,100
Gujarat	79,126	451.08	5,700
Haryana	21,483	260.09	12,100
Himachal Pradesh	9,315	44.17	4,700
Jammu and Kashmir	23,181	148.06	6,400
Jharkhand	33,385	179.43	5,400
Karnataka	39,373	344.63	8,800
Kerala	18,726	145.72	7,800
Lakshadweep	272	0.14	500
Madhya Pradesh	113,964	988.34	8,700
Maharashtra	134,261	1,239.09	9,200
Manipur	2,534	29.31	11,600
Meghalaya	3,477	15.94	4,600
Mizoram	738	3.94	5,300
Nagaland	1,677	8.83	5,300
Odisha	61,777	379.56	6,100
Puducherry	1,385	10.57	7,600
Punjab	46,407	434.7	9,400
Rajasthan	63,393	459.59	7,200
Sikkim	323	3.54	11,000
Tamil Nadu	56,601	334.25	5,900
Telangana	31,311	393.81	12,600
Tripura	7,731	60.89	7,900
Uttar Pradesh	207,635	2,081.72	10,000
Uttarakhand	21,191	103.95	4,900
West Bengal	66,291	451.18	6,800

Indiastat database (Lok Sabha unstarred question no. 2613, dated 16 December 2025) and Annual Report 2023–2024 Government of India DEPwD.

Fund utilization per beneficiary is calculated by dividing total funds utilized (INR million) by total ADIP beneficiaries in each state during the study period. All figures are reported in Indian Rupees (INR).

**Figure 2:**
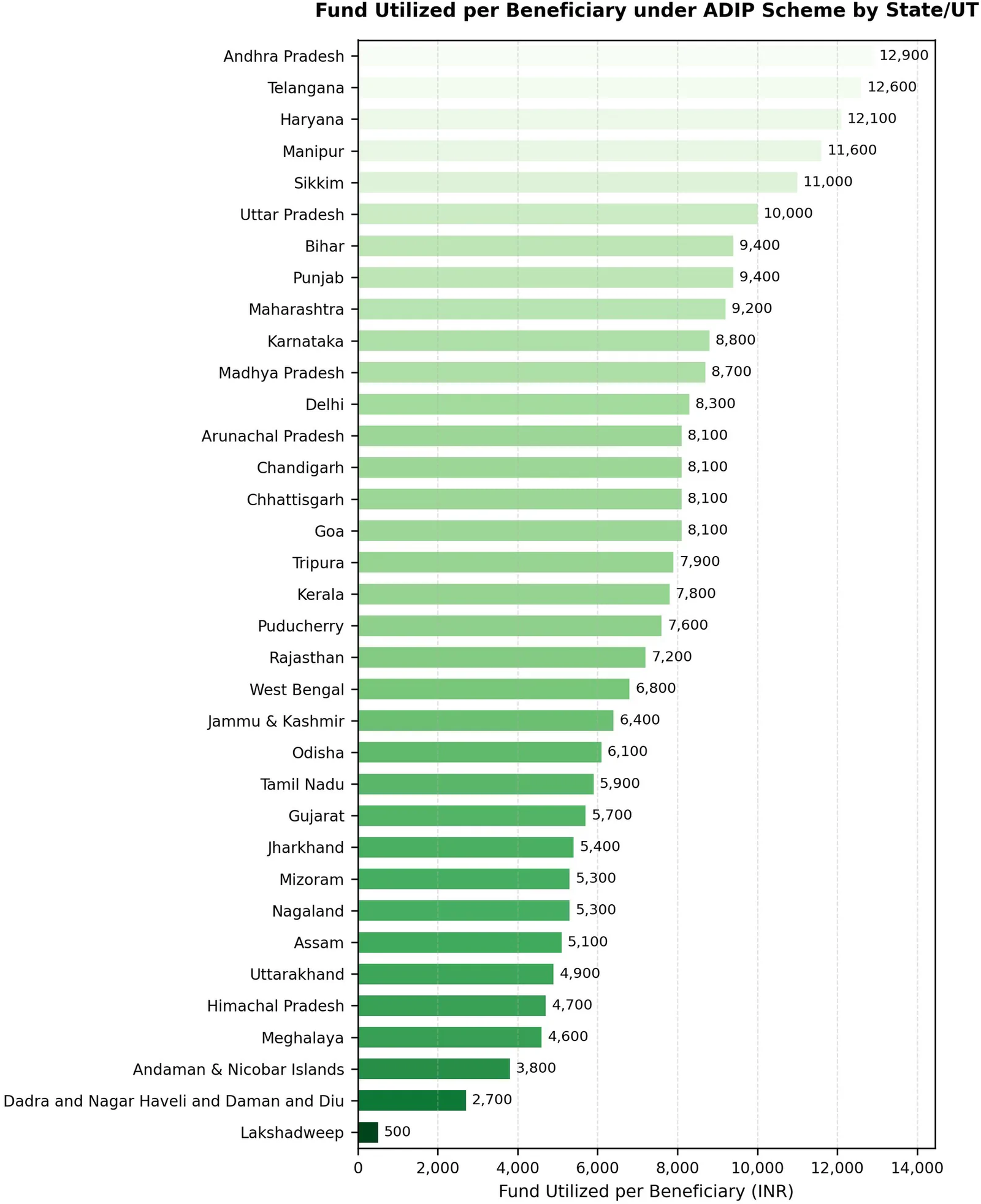
Fund utilized per beneficiary under the ADIP scheme by state and UT. Source: Indiastat database (Lok Sabha unstarred question no. 2613, dated 16 December 2025) and Annual Report 2023–2024 Government of India DEPwD.

Figure 2 provides a graphical presentation of interstate disparities in the financial dimension of the ADIP scheme. To measure the degree of financial disparity across states, the coefficient of variation and range ratio are calculated for fund utilization per beneficiary. The mean or average expenditure per beneficiary is ₹7,400, with a standard deviation of ₹2,800, resulting in a coefficient of variation of 0.38. This moderate variation suggests a comparatively low distribution relative to beneficiary coverage. Meanwhile, the range ratio of 25.8 reveals a stark utilization difference: Andhra Pradesh records the highest expenditure per beneficiary at ₹12,900, whereas Lakshadweep records the lowest at ₹500. The results indicate considerable variation in the financial utilization of the ADIP scheme across states.

### 3.4 Distribution camps and beneficiary outreach

To assess the beneficiary outreach through distribution camps, the average number of beneficiaries served per camp is calculated for each state/UTs in India from 2020 to 2024.

Table 4 presents state-wise camp distribution and beneficiaries reached per camp under the ADIP scheme from 2020 to 2024. The data indicate significant variations in the average number of beneficiaries covered by camps in India. Goa records the highest average number of beneficiaries per camp (471), followed by Maharashtra (358) and Andhra Pradesh (317). Uttar Pradesh, West Bengal, and Tamil Nadu also report a relatively high beneficiary outreach per camp. Comparatively low beneficiaries per camp are observed in Northeastern states such as Arunachal Pradesh (36), Manipur (29), Mizoram (27), and Sikkim (23). These differences suggest that the extent and scale of distribution camps vary across states, reflecting the differences in program implementation, population size, and geographic accessibility in the respective regions.

**Table 4:** State-wise distribution of ADIP camps and beneficiaries served per camp in India (2020–2024).

State/union territory	ADIP beneficiaries	ADIP camps	Beneficiary per camp
Andaman and Nicobar Islands	680	4	170
Andhra Pradesh	48,216	152	317
Arunachal Pradesh	715	20	36
Assam	55,036	467	118
Bihar	55,391	297	187
Chandigarh	1,795	15	120
Chhattisgarh	7,660	31	247
Dadra and Nagar Haveli and Daman and Diu	1,108	10	111
Delhi	16,140	126	128
Goa	2,827	6	471
Gujarat	79,126	388	204
Haryana	21,483	135	159
Himachal Pradesh	9,315	66	141
Jammu and Kashmir	23,181	169	137
Jharkhand	33,385	310	108
Karnataka	39,373	168	234
Kerala	18,726	154	122
Lakshadweep	272	1	272
Madhya Pradesh	113,964	718	159
Maharashtra	134,261	375	358
Manipur	2,534	88	29
Meghalaya	3,477	36	97
Mizoram	738	27	27
Nagaland	1,677	13	129
Odisha	61,777	448	138
Puducherry	1,385	14	99
Punjab	46,407	335	139
Rajasthan	63,393	262	242
Sikkim	323	14	23
Tamil Nadu	56,601	261	217
Telangana	31,311	183	171
Tripura	7,731	90	86
Uttar Pradesh	207,635	892	233
Uttarakhand	21,191	195	109
West Bengal	66,291	286	232

Indiastat database (Lok Sabha unstarred question no. 2613, dated 16 December 2025) and Annual Report 2023–2024 Government of India DEPwD.

Beneficiaries per camp are calculated by dividing the total number of beneficiaries by the total number of distribution camps organized in each state.

**Figure 3:**
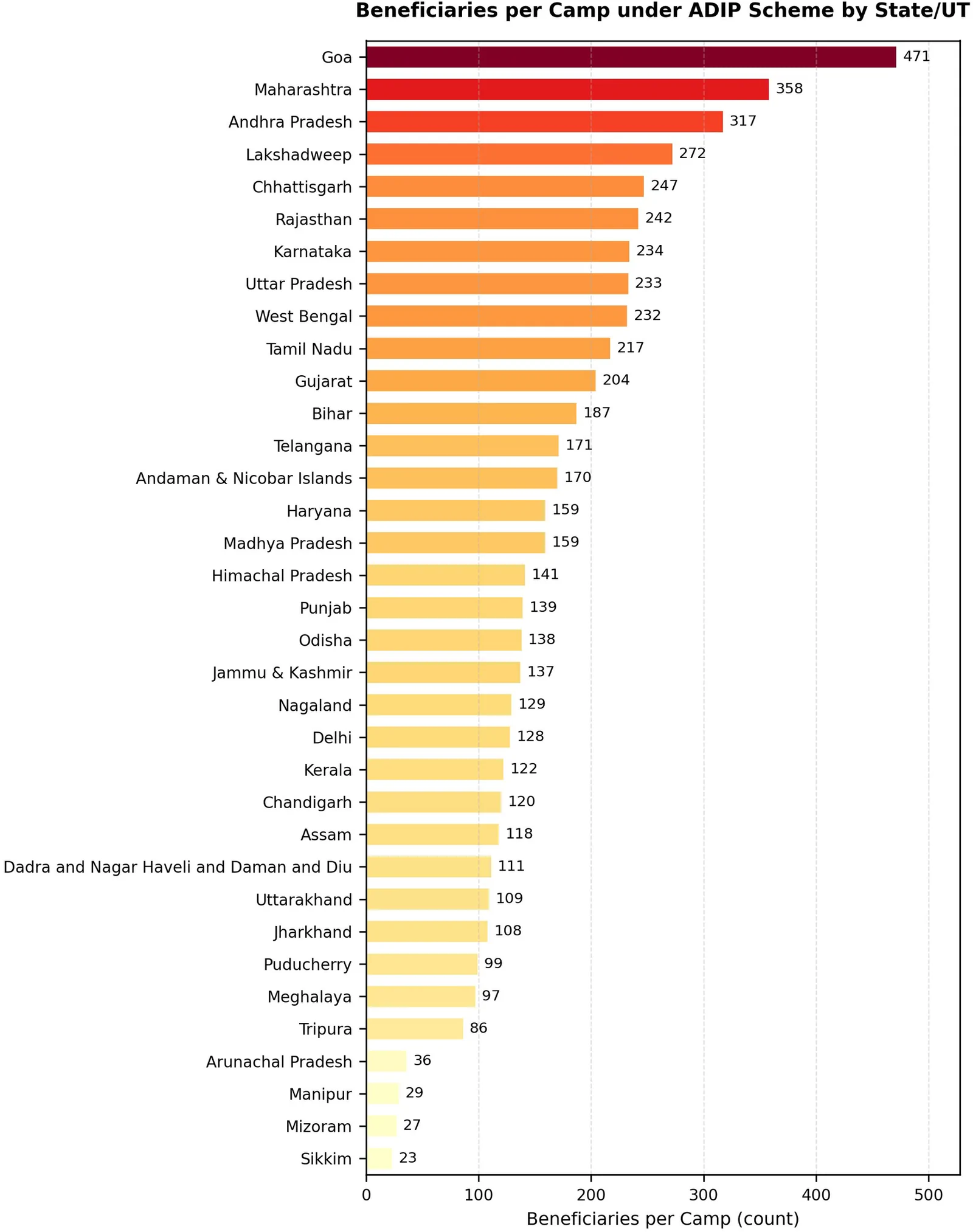
Beneficiaries per camp under the ADIP scheme by state and UT. Source: Indiastat database (Lok Sabha unstarred question no. 2613, dated 16 December 2025) and Annual Report 2023–2024 Government of India (33).

Figure 3 visualizes the variation in ADIP scheme performance across states and UTs by beneficiaries served per distribution camp. To measure the degree of variation in beneficiary outreach through camps across states, the coefficient of variation and range ratio are calculated for beneficiaries served per distribution camp. The mean value is 164.9 beneficiaries per camp, with a standard deviation of 95.05, resulting in a coefficient of variation of 0.58. This moderately high variability indicates notable interstate variations in the scale of camp-based distribution. The range ratio of approximately 20.5 indicates that the highest-performing state, which is Goa, with 471 beneficiaries per camp, serves more than 20 times the number served in Sikkim, with 23 beneficiaries, highlighting substantial differences in outreach capacity and program scale across states.

### 3.5 Findings

An analysis of publicly available data on the ADIP scheme reveals several interstate disparities in beneficiary coverage, financial utilization, and camp outreach. Beneficiary coverage relative to the disabled population varies across regions, which is evident from a CV of 0.65 and a range ratio of 16.4, indicating stark variation. Coverage is higher in small states and union territories, compared with large states with a larger disabled population. Financial utilization per beneficiary shows the highest disparity, with a range ratio of 25.8 and a coefficient of variation of 0.38, possibly due to the differences in type and cost of devices distributed across regions. With a CV of 0.58 and a range ratio of 20.5, camp-based distribution similarly reflects unevenness in operational capacity where the northeast states report lowest beneficiaries per camp. Taken together, these results indicate that implementation of the ADIP scheme is geographically uneven and not proportionately aligned with disability prevalence across states and union territories.

## 4 Discussion

The findings are interpreted in light of existing literature and their implications on policies related to assistive technology, disability rehabilitation, and service delivery. The interstate inequality in beneficiary coverage aligns with National Sample Survey data, which document disparities in access to disability support and rehabilitation services across sociodemographic and regional groups, reinforcing that geography remains a critical determinant of program outreach (21). Similarly, evidence (22) shows that the use of assistive devices among older adults is 71.27% in Goa, while it is 13.44% in Arunachal Pradesh, demonstrating the fundamental differences in state-level administrative and institutional capacity. The present findings support this pattern, where smaller union territories and states, particularly northeastern states, achieved a comparatively high coverage relative to their disabled populations, while large states with the greatest disability burden, such as Uttar Pradesh and Bihar, remained relatively underserved. Rashmi and Mohanty (27) found in their study that differences in geographic and socioeconomic aspects of disability prevalence affected both the demand and delivery of assistive services. States with dispersed rural populations faced compounded challenges in identifying eligible beneficiaries, coordinating distribution logistics, and sustaining outreach. Chauhan et al. (16) further noted that according to caregivers’ perspectives, bureaucratic inefficiencies and insufficient funding constrain the implementation of disability-related schemes, like ADIP, particularly in states where administrative systems are weaker. Therefore, we can understand that coverage gaps in the ADIP scheme reflect not only program shortfalls but also deeper inequalities in administrative capacity across regions.

Expenditure per beneficiary is shaped by the type and cost of assistive devices distributed, procurement process, and efficiency of the implementing agencies. Gupta et al. (28) observed that the delivery of wheelchairs by government programs showed considerable interstate disparities due to differences in state-level administrative mechanisms. States recording higher per-beneficiary cost may be distributing more sophisticated devices such as powered wheelchairs or advanced hearing aids, whereas states with lower expenditure may limit distribution to basic appliances. The absence of alignment between financial utilization and beneficiary coverage in our findings suggests that funding inputs alone do not determine program reach. Moreover, fragmented service systems are reflected in financial aspects and also affect the efficiency of assistive technology programs across states (29). According to another study (20), one of the challenges affecting the consistency of assistive technology distribution is inadequate financial allocations, alongside poor supply chain networks and weak regulatory enforcement.

Ground-level operationalization of the ADIP scheme is reflected directly in the number of beneficiaries served per distribution camp. The northeastern states, such as Manipur, Meghalaya, and Mizoram, report fewer beneficiaries per camp, a pattern that may be associated with geographic barriers, dispersed populations, and limited reachability. In contrast, states such as Goa, Maharashtra, and Andhra Pradesh record higher numbers of beneficiaries per camp, which may reflect the potential economies of scale associated with larger-scale camp organization and more cost-effective delivery. Brien et al. (30) support this interpretation, noting that barriers to access to assistive products in rural India are influenced by institutional capacity and local infrastructure, leading to low camp efficiency, especially in remote and hilly regions. Similarly, exceptional lessons with practical implications emerge from states such as Tripura and Assam, which have achieved high beneficiary coverage of their disabled populations by conducting several small camps rather than fewer large camps. Conducting large camps would maximize cost efficiency but risk the exclusion of beneficiaries in remote areas. However, smaller and more frequent camps may improve accessibility while requiring more administrative capacity and investment. Karki et al. (25) noted that assistive technology service distribution across Nepal, India, and Bangladesh suffered from major weaknesses such as limited user awareness and restricted service availability across quality indicators. Similar structural weaknesses that persist within India explain the camp-level disparities identified in this study.

As a consequence of poor regulatory enforcement, “Gray markets” have emerged, affecting regions differently based on their administrative oversight capabilities. States with stronger regulatory frameworks maintain better service quality and market standards (20). Senjam et al. (10) found that the unmet needs for assistive technology were disproportionately concentrated among older populations, rural residents, and persons with severe disabilities, which is the most vulnerable category, particularly in states with weaker service infrastructure. Similarly, Kulsum et al. (17) highlighted that implementation of the Rights of Persons with Disabilities Act, 2016 was hindered by factors such as limited awareness and accessibility gaps, impacting schemes like ADIP unevenly across states.

In the larger South Asian context, these findings gain further perspective. Badhal et al. (24) identified challenges— including affordability, availability, and government funding—in access to assistive technologies in the South Asian region. Tangcharoensathien et al. (26) suggest that to improve access to assistive technologies in low- and middle-income countries, policy interventions on both the supply and demand sides are necessary. The differences in access to assistive technologies across various states in India indicate that the task of expanding nationwide program coverage will require efforts directed at building more equitable state-level infrastructure to support successful implementation.

### 4.1 Limitations of the study

Several limitations in this study should be acknowledged. First, the figures for the disability population are drawn from the 2011 Census, which is now over a decade old and may not accurately represent the current scale or distribution of disability across states. The unavailability of more recent nationally representative disability data is a constraint that affects the precision of coverage estimates. Second, the cumulative nature of the beneficiary data poses challenges, as disaggregated data over the 5-year period are unavailable. It is possible that some individuals received multiple assistive devices during the study period, inflating coverage estimates. Third, the analysis does not accommodate state-level differences in device costs, as there would be considerable variations in the price of assistive devices, disallowing direct comparisons of per-beneficiary expenditure across states. Fourth, there is a constraint in the geographic unit of analysis as the study ignores within-state variation. Coverage that appears moderate at the state level may, in reality, be concentrated in a few districts, concealing deeper local inequalities. Finally, the descriptive nature of the analysis means that while patterns of variation can be identified, causal explanations for why certain states perform better than others cannot be established from this dataset alone.

### 4.2 Policy implications and recommendations

The study findings highlight several areas of concern where specific policy interventions are required. In states reporting the lowest level of beneficiary coverage relative to their disabled population—such as Chhattisgarh, Sikkim, and Bihar—a contextual and diagnostic approach is needed to identify whether gaps arise because of funding, implementation agency, awareness, or accessibility challenges. Conversely, the performance of states such as Goa, Maharashtra, and Tripura, which achieved high coverage or efficient camp organization, should be adapted as lessons for other states to improve implementation in underperforming regions. A closer scrutiny is needed for fund utilization per beneficiary, which shows sharp disparities across states. The ratio between the highest-spending state and the lowest-spending state is 25.8, raising questions about underutilization of funds or inefficiencies in implementing agencies, requiring deeper monitoring.

A related structural concern is the camp-based model through which assistive devices have been distributed under the ADIP scheme for over four decades. Although periodic camps allow low-cost bulk distribution, the wide interstate variation in beneficiaries served per camp demonstrates that reach remains limited, particularly in northeastern and geographically dispersed states. Rather than a uniform national reform, the findings support a hybrid, state-differentiated strategy: retaining camps where they already perform well, such as in Goa, Maharashtra, and Andhra Pradesh, while progressively integrating assistive device screening, prescription, recommendation, and referral into the primary healthcare delivery system—including Health and Wellness Centres under Ayushman Bharat and District Disability Rehabilitation Centres (DDRCs)—in states with low camp density. Embedding ADIP-linked services within routine healthcare delivery would allow continuous, need-based identification of beneficiaries and likely improve coverage where the camp model has consistently underperformed.

The reliance on the 2011 Census as the basis for estimating beneficiaries is a limitation that restricts the implementation of the ADIP scheme. As a priority, accurate, updated data at the district level are required for planning and distribution of disability rehabilitation services.

## 5 Conclusion

The ADIP scheme, operational since 1981 under the initiative of the Ministry of Social Justice and Empowerment, Government of India, was established to assist persons with disabilities in procuring high-quality and scientifically manufactured aids and appliances. This paper examined the implementation of the scheme across Indian states and UTs from 2020 to 2025, focusing on beneficiary coverage, fund utilization, and camp outreach. The findings gathered from secondary sources indicate that the scheme operates unevenly across regions. The most direct indicator, beneficiary coverage, reveals that larger states record fewer beneficiaries and disproportionately lower coverage of their disabled populations, raising concerns about need-based distribution strategies within the scheme. Data regarding fund utilization from each region show wide variations, indicating the underutilization and disproportionate distribution of funds allotted for the scheme. The operationalization of distribution camps also varies across regions, with northeastern states facing challenges of geographic isolation and limited infrastructure. To conclude, these three dimensions highlight the unequal distribution of services to people with disabilities across Indian states and UTs. Multiple factors—including administrative capacity, funding patterns, regional control of implementing agencies, and methods of identification and distribution—contribute to this unevenness. Addressing these would require unique targeted models based on the demographic needs of the region, with a special focus on regions where the coverage gap is widest.
